# Seasonal Adaptation of the Gut Microbiome in Japanese Macaques: Linking Gut Microbiome Shifts With Fermentative Function

**DOI:** 10.1002/ece3.72076

**Published:** 2025-09-01

**Authors:** Wanyi Lee, Tianmeng He, Yosuke Kurihara, Izumi Shiroishi, Kazunari Ushida, Sayaka Tsuchida, Goro Hanya

**Affiliations:** ^1^ Center for Ecological Research Kyoto University, Inuyama Campus Aichi Japan; ^2^ Japan Society for the Promotion of Science Tokyo Japan; ^3^ Primate Research Institute Kyoto University Aichi Japan; ^4^ Center for Education and Research in Field Sciences, Faculty of Agriculture Shizuoka University Hamamatsu Japan; ^5^ College of Bioscience and Biotechnology Chubu University Aichi Japan

**Keywords:** 16s rRNA sequencing, gut microbiome, Japanese macaques, *Macaca*, primate, seasonal variation

## Abstract

Seasonal fluctuations in food availability strongly influence the ecology of wild mammals, yet the role of the gut microbiome in mediating these challenges remains insufficiently explored. In this study, we examined how seasonal dietary shifts influence gut microbial composition and fermentative function in wild Japanese macaques ( *Macaca fuscata* ). Integrating meta‐16S rRNA sequencing and in vitro fermentation assays, we investigated how the gut microbiome and the associated fermentative ability of Japanese macaques vary with seasonal dietary shifts. Although alpha diversity remained relatively stable throughout the year, significant changes in microbial composition revealed a flexible, seasonally responsive microbiome. Importantly, in vitro fermentation assays indicated that fermentative ability was stable across seasons for leaf fermentation but flexible for fermenting easily fermentable monkey chow. This dual strategy may represent an essential adaptive feature of the macaque gut microbiome, balancing metabolic stability and plasticity to effectively cope with seasonal dietary fluctuations. By linking microbial dynamics with dietary variation, this study provides new insights into the feeding ecology of Japanese macaques and highlights the essential role of gut microbiomes in supporting the ecological success of temperate primates.

## Graphical abstract

Seasonal dietary shifts in Japanese macaques drive changes in gut microbiome composition and function. While overall diversity remains stable, microbial communities adapt by modulating fermentative processes, ensuring efficient and stable energy extraction from leaves while responding flexibly to non‐leaf foods.

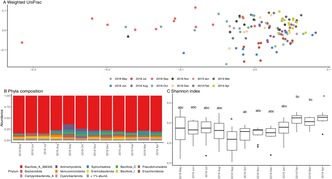

## 1 Introduction

By introducing periodic changes in climatic conditions and resource availability, season profoundly shapes the feeding ecology, behavior, and physiological adaptations of animals. To secure sufficient nutrients, mammals have evolved a range of behavioral and physiological strategies, including altered feeding behaviors, shifts in activity patterns, migration, and fat accumulation (Fryxell and Sinclair 1988; Mercer 1998; Marshall and Wrangham 2007; Hsiung et al. 2018). Increasingly, the gut microbiome is recognized as another vital mediator in how animals cope with spatially and temporally changing dietary and energy demands. This microbial community in the gastrointestinal tract plays a critical role in breaking down the otherwise indigestible fiber in plants. For nonruminant herbivores like the nonhuman primates, short‐chain fatty acids (SCFAs) derived from microbial fermentation can contribute 30%–60% of their energy requirements (Milton and McBee 1983; Popovich et al. 1997). Flexibility in the gut microbiome may enable mammals to buffer against energetic challenges posed by fluctuating food availability and quality, a necessity for survival in seasonally variable habitat.

An accumulating body of evidence from wild animals suggests that the gut microbiome exhibits remarkable flexibility in response to dietary variation. Seasonal or periodic food scarcity often compels animals to rely on fallback foods that are typically lower in nutritional quality (Marshall and Wrangham 2007). In such scenarios, individuals consuming low‐quality diets may possess a gut microbiome with enhanced digestive capabilities for low‐quality fallback foods, while high‐quality diets could also be reflected by the gut microbiome. For example, while wild wood mice transition from an insect‐rich diet in summer to a seed‐heavy diet in autumn, their gut community shifts significantly with fiber‐degrading Bacteroidetes like Alistipes enriched in abundance (Maurice et al. 2015). Alternatively, the gut microbiome may facilitate energy harvest during periods of food abundance. While the gut microbiome of the giant panda may be insufficient in providing energy during scarce seasons, as indicated by the lower fecal SCFA concentration, the panda gut microbiome during the shoot‐eating season enabled increased fat storage during fecal microbiota transplantation into a mouse model (Huang et al. 2022). The adaptability of the primate gut microbiome to seasonal dietary changes has been highlighted in black howler monkeys (Amato et al. 2015), where the howler gut microbiome produced more volatile fatty acid, a subset of SCFA, when the howlers reduced food intake due to high temperatures. Seasonal variation in the gut microbiome has been evident in other primate taxa, including sifakas (Springer et al. 2017), geladas (Baniel et al. 2021), capuchins (Mallott et al. 2018; Orkin et al. 2019), and apes (Gomez et al. 2016; Hicks et al. 2018). Such short‐term dietary shifts could even lead to convergence in gut microbiomes among different populations or species. During periods of fruit scarcity, gut microbiomes and metabolomes of western lowland and mountain gorillas converge, suggesting the shared reliance between the two species on certain gut microbial taxa for fiber digestion (Gomez et al. 2016). Collectively, these findings suggest that the gut microbiome plays a universal role in buffering mammalian hosts, including primates, against the energetic and nutritional challenges posed by seasonal environmental fluctuations.

The observed flexibility of the gut microbiome in response to dietary changes highlights the need for methods that can directly assess microbial functionality. In recent years, meta‐16S rRNA surveys, metagenomics, and metabolomic approaches have contributed valuable insights into how gut microbial communities respond to dietary variation. Yet, these methods remain largely indirect, focusing either on the presence of particular microbes or genes, or on the presence of metabolites after fermentation has occurred (Weinroth et al. 2022; Worsley et al. 2024). Meta‐16S rRNA analyses, for instance, mainly rely on the taxonomic composition to infer potential functionality, whereas metagenomics provides insights into the functional potential but could not directly assess whether or to what extent the functions are actually being activated. This is particularly relevant given that phylogenetically diverse taxa can perform similar fermentative functions (Tian et al. 2020) and that the fermentation process depends heavily on food properties (see review by Wang et al. (2019)). Likewise, metabolomics may show which end products are present but often lack information about the rate or efficiency of microbial fermentation processes (Worsley et al. 2024). Originally developed in animal science to evaluate feed digestibility and rumen function, in vitro fermentation assays now offer a valuable complement to molecular approaches by enabling direct observation of realized microbial function through the fermentation of an animal's actual dietary substrates. Using in vitro fermentation assays, the pioneering work of Lambert and Fellner (2012) demonstrated that primate species differ in microbial fermentation efficiency, with findings suggesting that such variation is shaped more by digestive anatomy and retention time of the primate species. As such, combining in vitro fermentation assays with ecological observations offers a powerful approach to investigate how gut microbes mediate host adaptation to dietary variability in the wild.

Our study subject, Japanese macaques ( *Macaca fuscata* ), represents an excellent system to explore the role of gut microbiome as an adaptive strategy to short‐term dietary variation, i.e., seasonality. Living at the northern limits of the nonhuman primate's global range, Japanese macaques inhabit a marginal habitat for primates. Compared to tropical forests, temperate forests are characterized by lower fruit production, as well as stronger but predictable seasonality (Hanya and Aiba 2010; Hanya et al. 2013). As high‐quality foods, fruits and seeds are preferentially consumed by the macaques whenever available. When fruits and seeds are scarce, the macaques feed on mature leaves and/or barks, depending on the region they inhabit (Tsuji 2010). Such a pattern is in contrast with the tropical primates, which tend to fall back to fig syconia and young leaves (Hanya et al. 2011; Tsuji et al. 2013). Accordingly, the temperate Japanese macaques face extended periods of food scarcity with fallback foods of lower nutritional value. While both tropical and temperate primates rely on the gut microbiome, the microbiome of temperate species may demonstrate enhanced function to cope with extreme dietary transitions. This may suggest the important yet undercharacterized role the gut microbiome may play in macaques' adaptation to the temperate region along with the strong seasonality.

Recent studies indicate that the gut microbiomes of Japanese macaques are influenced by both long‐term and short‐term dietary factors. For instance, the macaque gut microbiome composition is significantly shaped by the forest type they consistently inhabited (e.g., evergreen vs. deciduous), reflecting long‐term differences in food availability and quality across habitats (Hanya et al. 2020; Lee et al. 2023). Similarly, anthropogenic factors such as provisioning, crop‐raiding, or captivity over extended time alter what and how macaques eat in the long term, leading to sustained shifts in their gut microbiomes (Lee et al. 2019, 2023). A previous study focusing on seasonality, Sawada et al. (2022), recorded diet and collected fecal samples for three individual macaques inhabiting the evergreen forest of Yakushima Island and showed that a leaf‐based diet produced a more convergent gut microbial profile, distinct from the pattern when the animals' diet included seeds, fruit, or insects. While these studies reveal clear shifts in gut microbial composition, the functional implications of observed shifts remain underexplored. Noting the gaps, Hanya et al. (2020) tested fermentative ability via in vitro assays on the gut microbiome of frugivorous and folivorous populations respectively inhabiting the lowland and highland forests of Yakushima Island. The assay revealed the folivorous population having a significantly higher fermentative ability compared to more frugivorous populations. This raises important questions about whether this higher fermentative ability is a result of long‐term adaptations in the folivorous populations and whether the fermentative ability of a single population would be advanced or lost by the season‐specific diet.

Using an integrative approach, our study aims to provide a more comprehensive understanding of how the gut microbiome responds to seasonal dietary shifts at both the taxonomic and functional levels. We hope to move beyond descriptive microbiome studies and evaluate the microbiome's role in dietary adaptation. In particular, we conducted in vitro fermentation assays, aiming to provide direct insights into the functional capacity of the macaque gut microbiome. To complement this in vitro perspective, we also measured fecal SCFA levels as an indicator of in vivo fermentation, thereby assessing how microbial fermentation contributes to energy acquisition under different dietary conditions. In parallel, we used meta‐16S rRNA sequencing to link with gut microbial community dynamics, allowing us to examine whether certain dietary intake drives changes in both composition and function. We also aimed to identify microbial taxa that contribute to fermentative ability. Moreover, by monitoring gut microbiome composition both before and after the fermentation assays, we pinpointed the microbial taxa that are actively involved in the fermentation process. By investigating how gut microbiome maintains or adjusts their fermentative abilities across seasons, this research contributes to our understanding of the feeding ecology of Japanese macaques and offers broader insights into how primates have successfully radiated from tropical to temperate environments. By illuminating the specific mechanisms through which microbial communities enhance fermentative efficiency, this study contributes to a deeper understanding of how temperate mammals navigate energetic challenges under seasonally constrained conditions.

## 2 Methods

### 2.1 Fecal Sample Collection

**FIGURE 1:**
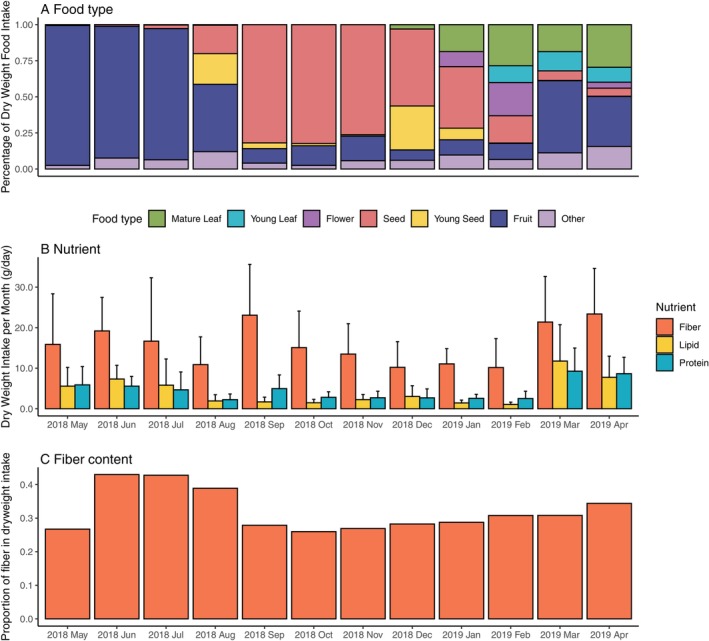
Seasonal variation in the (A) food intake, (B) nutrient intake, and (C) fiber content.

Fecal samples were collected from a well‐habituated group of Japanese macaques (Umi‐C) living in the lowland zone of western Yakushima, whose diet fluctuates substantially throughout the year (Hill 1997; Kurihara et al. 2020; Figure 1). The study was carried out from May 2018 to April 2019. At the beginning of the study, the Umi‐C group comprised 18 individuals (six mature females, five mature males, three juvenile females, and four juvenile males), with group membership fluctuating due to occasional emigration and immigration events; in total, 22 different individuals were observed. For a more detailed description of the study site and study subject, please refer to He et al. (2021). Within 30 min after defecation, we collected and stored the samples for molecular analysis in lysis buffer (0.5% SDS, 100 mM EDTA [pH 8.0], 100 mM Tris–HCl [pH 8.0], and 10 mM NaCl). Some individuals were sampled repeatedly throughout the study period, with an average of approximately five fecal samples collected per individual. Additionally, a portion of fecal samples was collected from unidentified individuals whose identity could not be confirmed at the time of collection. For the in vitro fermentation assay, we stored the feces in sealed plastic bags and removed as much air as possible. These feces were kept in a thermos jar filled with blue ice and then brought back to the field station within 8 h after collection.

### 2.2 Behavioral Observation

We conducted 1‐h focal sampling on individuals older than 12 months by continuous observation. The dataset comprised 1260 focal sampling sessions, averaging 57.3 sessions per individual. For feeding behavior, we recorded the species and the part eaten by the subjects. When direct counting was possible, we counted the number of food units consumed. A unit was operationally defined according to the food item (e.g., a leaf, a seed, or a fruit). We recorded feeding rates (units per second) when it was possible to continue direct counting for at least 60 consecutive seconds. The observational data were published on He et al. (2021).

For nutritional analysis of the food items, we conducted the sample collection 2.5–3 km from the home ranges of the study groups (outside of conservation areas). The samples were kept at −20°C in a freezer at the field station until they were brought to the Inuyama campus of Kyoto University for nutritional analysis. After we removed the portion that was not ingested by the focal animals, we dried the samples at 40°C until the weight became constant (for at least 24 h) using a vacuum incubator and weighed them to obtain dry weight per food unit (unit dry weight, g/unit). We measured crude lipid (CL) by the Soxhlet method with a diethyl‐ether solvent and neutral detergent fiber (NDF) by the method described in Van Soest et al. (1991). We determined crude protein (CP) as 6.25 times the total nitrogen content, where we measured the total nitrogen following the Kjeldahl method. Daily intake of food items and nutrients was estimated by multiplying the monthly energy intake rate (energy intake/observation time) by the monthly mean day length, then being log‐transformed. A total of 154 samples, including different parts of single plant species, were analyzed for nutritional value.

### 2.3 In Vitro Fermentation Assay

We conducted in vitro fermentation assays following the method described in previous studies on Japanese macaques (Hanya et al. 2020; He et al. 2023). Each gram of fecal sample was mixed with 4 mL of anaerobic McDougall's buffer (9.8 g NaHCO_3_, 2.44 g NaHPO_4_·2H_2_O, 0.57 g KCl, 0.47 g NaCl, 0.12 g MgSO_4_·7H_2_O, and 0.16 g CaCl_2_·2H_2_O in 1 L distilled water). To remove large particles, we squeezed the mixture through two layers of sterile gauzes. For the fermentation substrate, we used powdered *Eurya japonica* leaf and commercial monkey chow (AS, Oriental Yeast Co. Ltd.). The leaves were dried at 40°C and then ground by a mill (Wonder Blender WB‐1 Osaka Chemical) before use, while monkey chow was ground by a mill without further processing. We mixed filtrate with substrates in the ratio of 1 g filtrate to 0.01 g substrate, then poured it into a serum bottle. After replacing the headspace gas with 100% CO_2_, we closed the bottles with butyl rubber stoppers and aluminum seals. Bottles were placed in an oven set at 37°C with continuous shaking for 24 h. As indicators of fermentative ability, we measured fecal SCFA level, gas production, and SCFA production. Gas production was measured at 6, 12, 18, and 24 h of incubation by displacing a plunger of a 10‐mL glass syringe. For fecal SCFA level and SCFA production, we sampled 5 mL of the filtrate before and after 24‐h fermentation, centrifuged at 2600G for 5 min at room temperature, and then stored at –20°C.

### 2.4 SCFA Analysis

Analysis of SCFA was conducted at Chubu University. A portion of samples (1000 μL) was mixed with 100 μL of 12% HClO_4_, then centrifuged at 12,000 rpm for 10 min. The resultant supernatants were filtered through a 0.45 μm cellulose acetate membrane (Advantec, Toyo Roshi Kaisha Ltd., Japan). Samples were injected into an ion‐exclusion HPLC system with a SIL‐10 autoinjector (Shimadzu Seisakusho, Kyoto). The HPLC system consists of tandem ion‐exclusion columns (Shim‐pack SCR‐102H, 300 × 8 mm ID, 7 μm, Shimadzu) with a guard column (SCR‐102H, 50 × 6 mm ID). The mobile phase eluent is 5 mM p‐toluenesulfonic acid, and the post‐column reaction buffer is composed of 5 mM p‐toluenesulfonic acid, 0.1 mM EDTA•4H, and 20 mM BIS‐TRIS. The flow rate is set to 0.8 mL/min, and the column oven temperature is 40°C.

### 2.5 16S rRNA Amplicon Sequencing, Bioinformatic, and Statistical Analyses

After bead‐beating and centrifugation at 20,000× *g* for 1 min, we extracted DNA following the manufacturer's instructions using the QIAamp DNA Stool Mini Kit (QIAGEN, Hilden, Germany). We amplified the V3–V4 region of the 16S rRNA gene using primers 341F and 785R (Klindworth et al. 2013). After estimating DNA concentration using the Qubit dsDNA HS Assay Kit and a Qubit fluorometer (Thermo Fisher Scientific), the resulting PCR products were pooled in equal amounts (2 ng/sample), and paired‐end 300 bp sequencing was carried out on an Illumina Miseq platform (Illumina, San Diego, CA). We processed the raw sequences using QIIME2‐2024.5 (Bolyen et al. 2019). We implemented quality control, denoising, and chimera removal and generated the amplicon sequence variants (ASVs) using the DADA2 pipeline with default settings (Callahan et al. 2016). Taxonomy classification was performed with the Greengenes2 sequence database (2024.09 release), since Greengenes2 provides a unified resource that integrates 16S rRNA sequence and shotgun metagenomic data into a single reference phylogeny, facilitating more consistent and accurate taxonomic assignments (McDonald et al. 2024). Sequences classified as mitochondria, chloroplast, or singleton were excluded.

All statistical analyses were conducted using unrarefied data, as rarefaction has been shown to be statistically inefficient and can result in unnecessary loss of data (McMurdie and Holmes 2014). Statistical analyses were conducted in R version 4.4.1, with a significance level of 0.05. We implemented linear mixed models to compare alpha diversity indices, i.e., observed richness and Shannon's index, across months with individuals as a random factor. Permutational multivariate analysis of variance (PERMANOVA) was conducted for beta diversity, namely weighted and unweighted UniFrac distances. We assessed the significance of the intake of different food types and fiber content in explaining weighted and unweighted UniFrac distances by running distance‐based redundancy analysis (dbRDA) (Oksanen et al. 2019). Forward stepwise selection was employed to select the variable contributing the most to the variance. We assessed the significance of the intake of different food types and fiber content in explaining indicators of fermentative ability by running multiple linear mixed models, with individual and sampling month being the random factor. Our analyses revealed no strong collinearity among explanatory variables (maximum VIF = 2.1212). To identify key microbial genera involved in fermentation, we compared the gut microbiome composition before and after fermentation using Microbiome Multivariable Associations with Linear Models (MaAsLin 2) (Mallick et al. 2021) to find which taxa displayed a significant increase in relative abundance, with individual and sampling month being the random factor. Concurrently, we constructed microbial association networks employing the SparCC method using the R package *NetCoMi* (Peschel et al. 2021) based on a dataset containing genera representing at least 0.01% of the total read count. This integrative approach allowed us to model both positive and negative interactions among taxa, thereby providing insights into potential cooperative and competitive relationships that shape the microbial community as a functional unit.

### 2.6 Data Accessibility

Raw sequence data for the gut microbiome has been deposited in the DDBJ database under project PRJDB20590.

## 3 Results

### 3.1 Basic Profile of the Macaque Gut Microbiome

**FIGURE 2:**
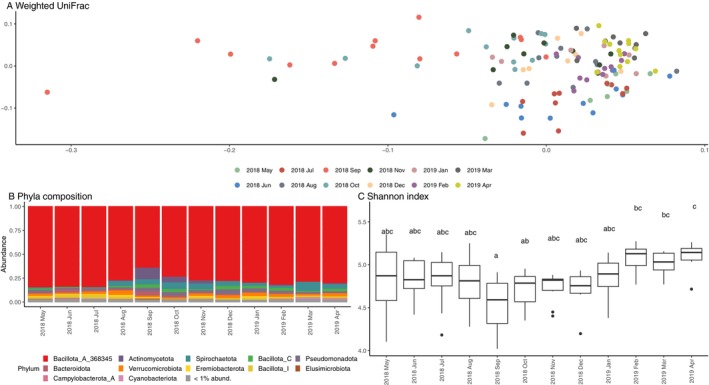
Seasonal variation in the macaque gut microbiome was evident in (A) the PCoA plot based on weighted UniFrac and (B) the compositional barplot at the phylum level, but not in (C) the Shannon index. Compact Letter Display (CLD) indicates results of post hoc pairwise comparison using estimated marginal means (emmeans package) with significance threshold at *p* < 0.05. Months sharing the same letter are not significantly different.

From 310 fecal samples of Japanese macaques, 28,879,934 high‐quality sequences of the 16s rRNA gene were clustered into 5627 ASVs after data preprocessing (Table S1). On average, each sample had 93,161 ± SD 123,521 reads, with 565 ± SD 240 ASVs (Figure S1). At the phylum level, the macaque gut microbiome was dominated by Bacillota_A (78.54% ± SD 8.57%), followed by minor phyla including Actinomycetota (7.09% ± SD 7.23%), Spirochaetota (5.89% ± SD 3.68%), and Bacillota_C (3.30% ± SD 2.29%) (Figure 2B). At the family level, the top 3 dominant families were Lachnospiraceae (24.47% ± SD 8.53%), Ruminococcaceae (15.03% ± SD 4.99%), and Oscillospiraceae (14.04% ± SD 5.04%) (Figure S2B).

### 3.2 Seasonal Variation in the Macaque Diet, Nutrient Intake, and Gut Microbiome

The diet of Japanese macaques shifted from fruit‐dominant (May–July) to seed‐dominant (August–December), then to a mixed diet incorporating leaves (December–April), with corresponding changes in nutrient intake (Figure 1A,B). In general, intake of fiber, protein, and lipid was highly correlated to each other, with Pearson's correlation coefficient *r* ranging from 0.6864 to 0.9200 (Figure S3). Fiber content as a percentage of total dry matter was highest from June to August 2018, ranging from 38.9% to 43.0%, and remained around 29% (range: 26.0%–30.8%) for the other months (Figure 1C). Both observed richness and the Shannon index showed no clear seasonal trend, with most months exhibiting statistically indistinguishable alpha diversity values (linear mixed‐effects model; Figure 2C; Table 1; Figure S1).

**TABLE 1:** Seasonal variation in observed ASV richness and Shannon diversity.

Month	Observed			Shannon		
	Mean	SE	CLD	Mean	SE	CLD
2018 May	393.66	35.30	ab	4.81	0.12	abc
2018 June	412.40	27.76	ab	4.82	0.09	abc
2018 July	367.55	27.75	ab	4.80	0.09	abc
2018 August	353.97	23.59	a	4.80	0.08	abc
2018 September	413.58	27.76	ab	4.53	0.09	a
2018 October	359.91	26.11	ab	4.70	0.09	ab
2018 December	339.04	24.76	a	4.71	0.09	abc
2018 November	357.46	27.75	ab	4.73	0.08	abc
2019 January	355.70	27.76	ab	4.84	0.09	abc
2019 March	400.67	26.14	ab	5.00	0.09	bc
2019 February	412.62	27.73	ab	5.08	0.09	bc
2019 April	476.14	24.77	b	5.10	0.08	c

*Note:* Mean ± standard error (SE) are reported for each metric. Compact letter display indicates results of post hoc pairwise comparisons using estimated marginal means with a significance threshold at *p* < 0.05. Months sharing the same letter are not significantly different.

**FIGURE 3:**
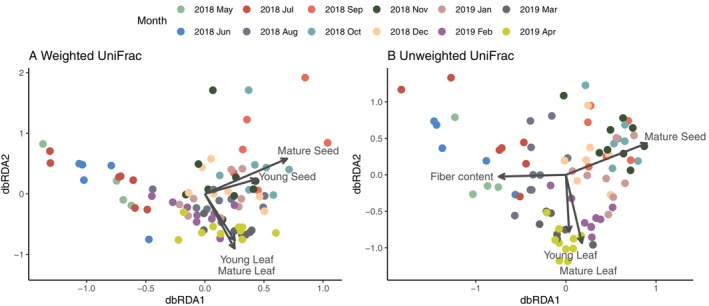
Distance‐based redundancy analysis (dbRDA) ordination showing how macaque gut microbial community structure varies with dietary components. Each point represents the microbial composition of an individual sample, calculated using (A) weighted and (B) unweighted UniFrac distances. Arrows depict dietary variables significantly influencing the gut microbiome (forward‐selected according to the adjusted *R* ^2^‐value criterion), with arrow length proportional to the strength of the association.

Although alpha diversity varied minimally, beta diversity displayed notable seasonal changes, with samples clustering by month (Weighted UniFrac: *R* ^2^ = 0.3855, *p* = 0.001; Figure 2A; Unweighted UniFrac: *R* ^2^ = 0.2891, *p* = 0.001; Figure S4). To investigate how macaque gut microbiome composition is influenced by dietary intake, we conducted dbRDA with forward selection. Due to high collinearity among lipid, fiber, and protein, only fiber content as a percentage of total dry matter intake was included in the dbRDA. dbRDA of the full model showed that dry weight intake of fruit, mature leaf, young leaf, mature seed, and young seed significantly influenced weighted UniFrac (adjusted *R* ^2^ = 0.1742; Table S2). Among these factors, forward selection identified dry weight intake of mature seed, mature leaf, young seed, and young leaf as the most important variables (adjusted *R* ^2^ = 0.1709; Table 2). Biplot scores indicated that mature seed and young seed intake are major drivers of variation along the dbRDA axis 1, whereas mature and young leaf intake primarily influence dbRDA axis 2 (Figure 3A). For unweighted UniFrac, dry weight intake of fruit, mature leaf, mature seed, and fiber content were the significant factors (adjusted *R* ^2^ = 0.1465; Table S2). Forward selection identified dry weight intake of mature leaf, mature seed, fiber content, and young leaf as the most important variables explaining variation in unweighted UniFrac metrics (adjusted *R* ^2^ = 0.1390; Table 2). Biplot scores indicated that seed intake and fiber content are the major contributors of dbRDA axis 1, while mature leaf and young leaf intake are aligned with dbRDA axis 2 (Figure 3B).

**TABLE 2:** Results of forward selection in the dbRDA analysis in weighted and unweighted UniFrac.

	Variable	Weighted UniFrac		*p*	Variable	Unweighted UniFrac		*p*
		AdjR2Cum	Pseudo‐*F*			AdjR2Cum	Pseudo‐*F*	
Food item	Mature seed	0.0706	7.6109	0.002**	Mature seed	0.0700	7.5439	0.002**
	Mature leaf	0.1271	6.5683	0.002**	Mature leaf	0.1228	6.1784	0.002**
	Young seed	0.1503	3.3182	0.002**	Fiber proportion	0.1318	1.8851	0.012*
	Young leaf	0.1709	3.0839	0.004**	Young leaf	0.1390	1.6965	0.020*

*Note:* Each variable is displayed in the order in which it was selected. *F*, *p*, and AdjR2Cum values are displayed. None of the selected variables showed significant multicollinearity (VIF ranges from 1.0825 to 15.4151). Asterisks denote significance level (*p* < 0.05 = *, *p* < 0.01 = **, *p* < 0.001 = ***).

### 3.3 Functional Response of Gut Microbiome to Seasonal Diet

**FIGURE 4:**
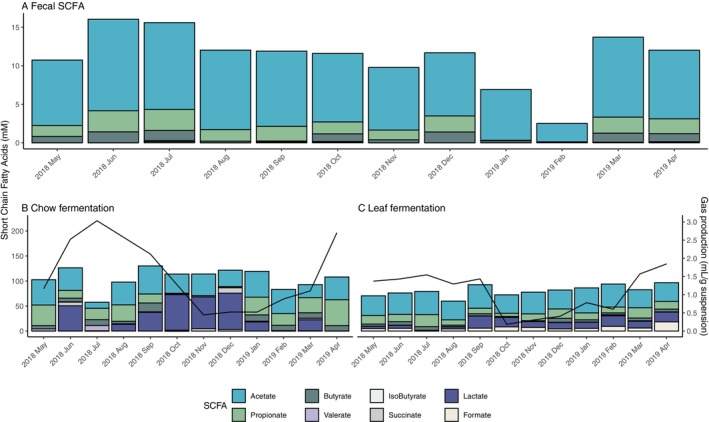
Seasonal variation in fecal and in vitro fermentation short‐chain fatty acid (SCFA) concentrations in Japanese macaques. (A) Fecal SCFA concentrations (mM) across months. (B) SCFA production and gas production during in vitro fermentation with chow. (C) SCFA production and gas production during in vitro fermentation with leaf.

We observed substantial fluctuations in total fecal SCFA levels throughout the sampling months (Kruskal–Wallis chi‐squared = 50.745, *p* < 0.001, Kruskal–Wallis rank sum test; Figure 4A; Table S3). A linear mixed‐effects model was used to examine the effect of dietary components on the response variable. The linear mixed model (marginal *R* ^2^ of 0.2597) revealed a significant negative effect of mature leaf (estimate ± SE = 0.13 ± 0.47) and a significant positive effect of fruit (estimate ± SE = 1.58 ± 0.44) (Table 3).

**TABLE 3:** Fixed effects estimates from linear mixed models for indicators of fermentative ability.

		Estimate (*β*)	SE	df	*t*	*p*
Fecal SCFA level	Mature leaf	−1.61	0.60	38.94	−2.67	0.011*
	Young leaf	0.64	0.69	86.91	0.92	0.360
	Mature seed	0.13	0.47	48.48	0.28	0.783
	Young seed	0.30	0.69	55.70	0.43	0.669
	Fruit	1.58	0.44	82.56	3.58	0.001***
	Flower	0.00	1.18	88.86	0.00	0.998
	Other	0.71	0.58	88.91	1.23	0.221
	Fiber content	8.96	12.46	39.79	0.72	0.476
SCFA production (leaf fermentation)	Mature leaf	7.43	2.72	90.00	2.73	0.008**
	Young leaf	3.01	3.46	90.00	0.87	0.387
	Mature seed	3.67	2.03	90.00	1.81	0.074
	Young seed	−1.76	2.93	90.00	−0.60	0.550
	Fruit	−1.57	2.20	90.00	−0.72	0.476
	Flower	−5.64	6.33	90.00	−0.89	0.375
	Other	−2.23	3.12	90.00	−0.71	0.477
	Fiber content	18.15	54.50	90.00	0.33	0.740
Gas production (leaf fermentation)	Mature leaf	0.06	0.08	24.83	0.74	0.465
	Young leaf	0.09	0.10	61.60	0.95	0.345
	Mature seed	−0.09	0.06	19.45	−1.47	0.157
	Young seed	−0.02	0.09	20.07	−0.26	0.795
	Fruit	0.16	0.06	47.62	2.64	0.011*
	Flower	0.08	0.17	89.44	0.49	0.623
	Other	0.22	0.09	89.40	2.56	0.012*
	Fiber content	−0.47	1.60	18.92	−0.29	0.774
SCFA production (chow fermentation)	Mature leaf	−11.92	10.16	24.05	−1.17	0.252
	Young leaf	16.78	10.32	24.95	1.63	0.116
	Mature seed	5.70	4.20	16.92	1.36	0.193
	Young seed	3.69	6.36	9.16	0.58	0.576
	Fruit	2.50	4.74	13.09	0.53	0.607
	Flower	52.80	44.88	25.00	1.18	0.251
	Other	−5.24	7.76	23.91	−0.68	0.506
	Fiber content	4.48	132.61	18.53	0.03	0.973
Gas production (chow fermentation)	Mature leaf	−0.26	0.34	23.33	−0.77	0.450
	Young leaf	0.29	0.37	28.63	0.79	0.435
	Mature seed	−0.02	0.16	19.32	−0.15	0.884
	Young seed	0.01	0.27	7.13	0.03	0.980
	Fruit	−0.01	0.18	17.54	−0.07	0.945
	Flower	−0.23	0.92	22.75	−0.25	0.809
	Other	0.08	0.25	26.96	0.33	0.748
	Fiber content	6.79	4.77	16.38	1.42	0.174

*Note:* Asterisks denote significance level (*p* < 0.05 = *, *p* < 0.01 = **, *p* < 0.001 = ***).

The result of the in vitro fermentation assay was intriguing, highlighting differences between substrate types and their fermentation dynamics. Overall, SCFA and gas production were higher during chow fermentation (SCFA: Kruskal–Wallis chi‐squared = 37.752, *p* < 0.001; Gas: Kruskal–Wallis chi‐squared = 14.503, *p* < 0.001, Kruskal–Wallis rank sum test production). Pearson's correlation test revealed that total SCFA and gas production weakly correlated with each other when leaf was the substrate (*r* = 0.2301, *p* = 0.01), but this pattern was not found when chow was the substrate (*p* > 0.05).

When using leaves as the fermentation substrate, both SCFA production and gas production varied significantly by month but were explained by different dietary factors (SCFA: Kruskal–Wallis chi‐squared = 15.612, *p* = 0.0272; Gas: Kruskal–Wallis chi‐squared = 60.851, *p* < 0.001; Figure 4C). The linear mixed‐effects model for SCFA production during leaf fermentation revealed that the dry weight intake of mature leaf (estimate ± SE = 7.44 ± 2.72) had a statistically positive effect (marginal *R* ^2^ = 0.1869, Table 3). By contrast, for gas production during leaf fermentation, young seed had a significant negative effect (estimate ± SE = −0.02 ± 0.09), whereas Other had a significant positive effect (estimate ± SE = 0.22 ± 0.09) (marginal *R* ^2^ = 0.3517, Table 3). When using chow as substrate, only gas production differed significantly by month (SCFA: Kruskal–Wallis chi‐squared = 15.612, *p* = 0.1562; Gas: Kruskal–Wallis chi‐squared = 33.804, *p* < 0.001; Figure 4B). According to the results of the linear mixed model, none of the factors significantly influenced SCFA production and gas production (Table 3).

### 3.4 Microbial Response During Fermentation

**FIGURE 5:**
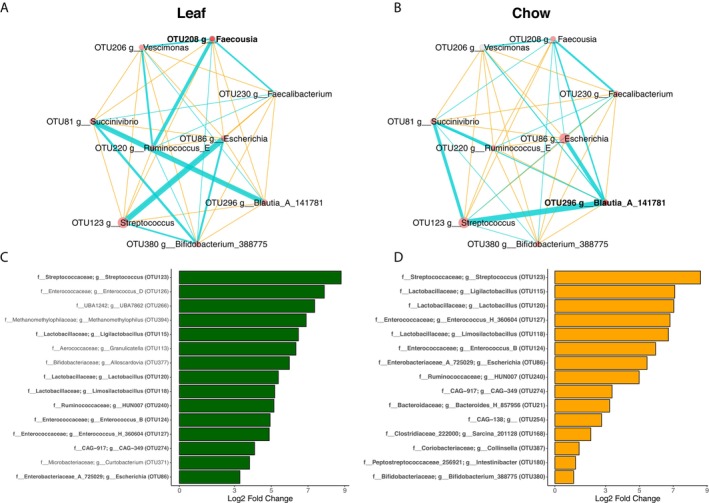
Network analysis of macaque gut microbiome after (A) leaf fermentation and (B) chow fermentation using SparCC correlation coefficients. The figure shows networks between genera whose number of reads are at least 1% of the total number of reads. Node color represents the cluster nodes belong to; node size represents the centered log‐ratio transformed counts of the genera. The edges represent the correlation coefficients between genera, with the color orange for negative correlations and blue for positive correlations. Nodes of networks are shown when their absolute correlation coefficients are greater than 0.3 (C, D). Bar plots displaying the top 15 genera whose relative abundance significantly increased after fermentation using (C) leaf and (D) chow as substrate. Genera were identified using Maaslin2 by comparing gut microbiomes before and after fermentation. Bolded font indicate genera commonly found between C and D.

To identify key microbial genera involved in fermentation, we compared the gut microbiome composition before and after fermentation to find taxa that displayed a significant increase in relative abundance. Despite employing distinct fermentation substrates (leaf powder versus monkey chow), the resulting microbial communities exhibited increases in a notably similar subset of genera. Specifically, fermentation of leaf powder enriched 39 genera spanning 24 families, including *Streptococcus*, *Enterococcus*, *UBA7862*, *Methanomethylophilus*, and *Ligilactobacillus* (Figure 5C). Using monkey chow, we observed abundance significantly increased in 21 genera from 16 families, with Lactobacillaceae being the most represented family (Figure 5D). The top five genera included *Streptococcus*, *Ligilactobacillus*, *Lactobacillus*, *Enterococcus H 360604*, and *Limosilactobacillus*. Observation of the top 15 increased genera showed that similar genera were responsible for leaf and chow fermentation. The two conditions shared 20 increased genera, while 19 exclusively increased when using leaf and 1 exclusively increased with chow.

Network analysis also showed a considerable overlap in structure and membership in both conditions (ARI = −0.125, *p* > 0.05; GCD = 0, *p* > 0.05; Figure 5A,B). The SparCC correlation network for leaf fermentation is comprised of one node and one cluster of eight nodes (nine nodes in total), with *Faecousia* serving as the hub. Among the nodes in networks, we found four, namely *Streptococcus*, *Escherichia*, *Bifidobacterium*, and *Succinivibrio*, that were also identified by Maaslin2 as differentially abundant. The network for chow fermentation similarly included one node and one cluster of eight nodes, but in this case, *Balutia A* served as the hub. By Maaslin2, only three nodes, namely *Escherichia*, *Bifidobacterium*, and *Streptococcus*, were identified as differentially abundant using samples from chow‐based fermentation.

## 4 Discussion

### 4.1 Functional Flexibility of the Macaque Gut Microbiome

Our study offers insights into the plasticity of gut microbial function, particularly in terms of SCFA production, as a buffer against the energetic challenges posed by seasonal dietary fluctuations for wild animals. Since SCFA and gas production are only weakly correlated in chow fermentation and not correlated in leaf fermentation, these two processes may simply be regulated by different microbial taxa and metabolic pathways (e.g., acetogenesis vs. methanogenesis). To understand the contribution of the gut microbiome to the host, we mainly discuss the changes in SCFA production. For SCFA production during leaf fermentation, we found a significant positive effect from the intake of mature leaf, suggesting that the intake of mature leaf did select for microbes better at fermenting leaf. This finding diverges from our initial hypothesis that fiber content would be the primary determinant of microbial function. One possible explanation is that this effect is confounded by the highly fibrous fruits, *Machilus thunbergia* (NDF 45.8%) and *Ficus superba* (NDF 45.1%), whose fiber content is comparable to the mature leaves (NDF 43.1 ± 11.9) and higher than other fruits (NDF 32.5 ± 11.6). From June to August, *M. thunbergia* and *F. superba* together accounted for 83.11%, 71.31%, and 36.43% of the macaque's dry weight intake. Moreover, mature leaf consumption may select for microbes capable of metabolizing plant secondary metabolites rather than the fiber‐degrading microbes. This interpretation is supported by Kohl et al. (2014), who demonstrated that woodrats consuming a phenolic‐rich diet harbored gut microbiota with an increased abundance of genes involved in the metabolism of aromatic compounds. Furthermore, when woodrats were treated with antibiotics, they exhibited significant weight loss when consuming the phenolic‐rich diet, highlighting the critical role of gut microbiota in facilitating detoxification and enabling energy extraction from chemically defended plant material.

In contrast, chow fermentation showed a different pattern, wherein none of the factors was statistically significant. Given that easily‐ fermentable substrates like chow could be digested without specialized metabolic pathways, the absence of significant predictors suggests that multiple microbial taxa could contribute to fermentation. However, the degree of plasticity varies according to the fermentation substrate—leaf fermentation remained relatively stable (1.62‐fold difference between highest and lowest SCFA production), whereas chow fermentation showed much greater variability (2.28‐fold difference). This distinction suggests that gut microbial fermentation is context‐dependent, with different adaptive roles for leaf and non‐leaf foods. Thus, we propose that the fermentative plasticity observed for non‐leaf foods is a result of microbial functional redundancy, wherein multiple taxa can perform similar metabolic roles, leading to variable SCFA production depending on the dominant microbial groups at a given time. Meanwhile, the stability of leaf fermentation suggests a conserved microbial function that ensures reliable energy extraction from fiber‐rich resources. This dual strategy may represent a key adaptive feature of the macaque gut microbiome, balancing metabolic stability and flexibility in response to seasonal dietary fluctuations.

This dual strategy may be closely linked to the presence of core fermentative microbes that remain consistently active across different substrates and seasons. These microbes likely play a key role in maintaining SCFA production, ensuring stability for leaf foods while allowing for adaptive responses to non‐leaf foods. These taxa include genera such as *Streptococcus*, *Ligilactobacillus*, *Lactobacillus*, *Faecalibacterium*, and *Bifidobacterium*, which are known for their role in fermentation. For example, *Bifidobacteria* ferment various oligosaccharides to produce lactate and acetate, which promote butyrate‐producing microbes by cross‐feeding (Belenguer et al. 2006); *Streptococcus* and *Ligilactobacillus* are known to predominantly produce lactic acid (du Toit et al. 2014; Zheng et al. 2020). By retaining this functional core, Japanese macaques may benefit from a reliable output of SCFAs for non‐leaf foods even when there is an abrupt dietary switch. Nevertheless, due to the limited research exploring the enriched microbes during fermentation for wild animals, it is hard to conclude whether core fermentative microbes are unique to Japanese macaques or would be applicable to other species. However, we did find in Hanya et al. (2020), that *Streptococcus* was also enriched during an in vitro fermentation assay using feces of both frugivorous and folivorous populations, revealing a potentially important role this genus may have for Japanese macaques. Similarly, in one study using human feces, *Bifidobacterium* and *Ruminococcus* were repetitively found enriched during the fermentation of multiple prebiotics (Fehlbaum et al. 2018). Future research should focus on identifying the specific mechanisms that underpin this functional resilience and plasticity, such as the enzymatic pathways shared among the core microbes.

Our study offers valuable insights into the context for folivory‐induced enhancement in fermentative function. The degree of folivory‐induced enhancement may depend on the necessity for microbial plasticity among different populations. The frugivorous population of Japanese macaques in Yakushima, our study subject, inhabits one of the most fruit‐rich temperate forests within the distribution of nonhuman primates (Hanya and Aiba 2010). Given that mature leaves constitute less than 30% of their monthly diet and only 5.04% of their annual feeding time, the folivory‐induced enhancement we observed may be relatively weak. Especially in the study year, the proportion of fruits and seeds in the macaque diet during winter and spring appeared relatively high compared to previous studies (Agetsuma 1995; Hill 1997; Kurihara et al. 2020). In years with lower fruit availability, as described in previous studies, selective pressures on fiber‐degrading microbial function may be even stronger. A direct comparison by Hanya et al. (2020) between frugivorous and folivorous macaque populations on Yakushima Island further supports our hypothesis that folivory selects for more efficient fermentation of leaves. Significantly higher gas production and lower pH after fermentation were observed during in vitro fermentation assays using feces from the folivorous population, indicating their higher microbial activity than the frugivorous population. The greater reliance on folivory in the folivorous population, with leaves comprising 38.2% of their annual feeding time (Hanya 2004), likely imposes stronger selective pressures that favor microbial adaptations for enhanced leaf degradation. However, since Hanya et al. (2020) was conducted in May, the effect of folivory on microbial function may also be obscured by the intake of fibrous fruits as aforementioned. While shedding light on the potential of gut microbial function to adapt to folivory, our study underscores the need for further research on gut microbial plasticity in macaques experiencing more pronounced seasonal shifts or limited access to high‐quality food sources in different habitats or years. It has been revealed that forest type strongly shaped the diet and gut microbiome composition of Japanese macaques (Tsuji 2010; Lee et al. 2023). In addition to the altitudinal difference, research comparing populations living in more divergent environments and seasons may reveal clearer patterns.

### 4.2 Comparison With the Tropical Primates

Ecological differences between temperate and tropical primates influence how gut microbiomes may buffer their host against energy limitation, as seen in comparisons between Japanese macaques and howler monkeys ( *Alouatta pigra*), a tropical primate species well‐studied for its gut microbiome dynamics. While also varying across seasons, the gut microbiome of howler monkeys produced more fecal volatile fatty acid in response to reduced energy intake (Amato et al. 2015). In contrast, the low fecal SCFA level of Japanese macaques was associated with reduced energy intake due to the intake of mature leaves. This fundamental difference in fermentative ability reflects the contrasting underlying causes for their energy limitation. Howler monkeys face energy constraints primarily due to high maximum daily temperatures, which suppress overall food intake, but food sources of high nutritional quality remain available. For example, the howler's diet is composed of 50.4% ripe fruits during the most energy‐limiting dry‐fruit‐dominated period (Amato and Garber 2014). Our study also revealed a positive effect of fruit intake on fecal SCFA level and a higher SCFA production during fermentation using chow than leaf. Similarly, fecal SCFA levels of giant pandas were also higher when the animals fed on shoots instead of leaves (Huang et al. 2022). Hence, the enhanced fermentative ability may be facilitated by the howlers' reliance on more easily fermentable fruits. These findings emphasize the close relationship between fermentative outcomes and ingested foods, as readily fermentable substrates enhance microbial activity, while low‐quality substrates, such as mature leaves, present challenges for efficient fermentation. The contrasting pattern observed in temperate Japanese macaques and tropical howler monkeys suggests that ecological context strongly influences gut microbiome functionality. Our study warrants more cross‐species studies examining microbiome composition, fermentation efficiency, and SCFA profiles in wild primates across different climatic zones.

### 4.3 Interpreting In Vitro Fermentation Assay in Ecological Context

While in vitro fermentation assays have emerged as another valuable tool for studying the functional capacity of mammalian gut microbiomes, the ecological interpretation of this method requires careful consideration of their limitations. Most importantly, the controlled nature of these assays may not fully replicate the complexity of in vivo gut environments, where microbial activity is dynamically influenced by factors such as continuous food influx, pH gradients, host‐derived enzymes, and oxygen levels. For example, even brief exposure to suboptimal anaerobic conditions during sample collection and preparation may inhibit obligate anaerobes, leading to an underestimation of their contributions to fermentation processes. Additionally, there is a possibility that the core fermentative microbes identified in these assays primarily represent taxa that grow the fastest under laboratory conditions, rather than those that play a dominant or ecologically consistent role in vivo. This bias toward fast‐growing microbes may obscure the contributions of slow‐growing taxa, some of which specialize in breaking down complex polysaccharides or secondary metabolites. Such microbes, despite their critical role in digesting low‐quality foods, may be underrepresented in short‐term assays, while opportunistic microbes that readily metabolize simple sugars may dominate, potentially skewing conclusions about microbial functionality.

Our study revealed inconsistencies between gas and SCFA production, suggesting that these two processes do not always align. Larger gas production likely indicates high activity of certain microbial groups, but these may not be the same as those primarily responsible for SCFA production. SCFAs play a direct role in host energy metabolism by serving as a key energy source, whereas gas production primarily represents metabolic byproducts of microbial fermentation. Although the exact mechanism remains unclear, the microbial taxa involved in gas production may still influence gut microbial dynamics through cross‐feeding interactions, pH modulation, and other processes that shape the overall fermentation environment. Careful examination of multi‐omics data, including metagenomics and metabolomics, could help disentangle these overlapping metabolic processes and provide a clearer picture of microbial fermentation both in vitro and in vivo. Our study highlights the potential of in vitro fermentation assays, with further refinement and integration of multi‐omics approaches, to provide a more comprehensive understanding of gut microbiome function and its role in host adaptation.

In addition, fermentation time may influence the extent of microbial fermentation. In our in vitro fermentation experiments, a fixed fermentation time, i.e., 24 h, may have restricted the microbe's capacity to extract energy from fibrous foods, highlighting the interplay between time constraints and substrate fermentability. For Japanese macaques, the average food retention time on a high‐fiber diet (NDF 37.5%) is approximately 35.1 h (Sawada et al. 2011). This retention time is comparable to that of tropical macaques ( *Macaca fascicularis* , 36.9 h), guenons (*Cercopithecus* spp., 16–34 h), and chimpanzees (31–48 h), but significantly shorter than orangutans (73–124 h) and gorillas (57–87 h), whose larger gut size allows for prolonged fermentation (Lambert 2002; Remis and Dierenfeld 2004; Chang et al. 2016). A shorter retention time imposes constraints on the degree of substrate degradation during fermentation, particularly for fiber‐rich, leaf‐dominated diets. Even at maximum microbial activity, the substrates may not be fully digested within the available time, resulting in limited SCFA production from the low‐quality foods. These limitations revealed an important aspect of microbial fermentation—the balance between substrate fermentability, microbial capacity, and fermentation time.

## 5 Conclusions

Our study highlights the ecological significance of gut microbial plasticity in primate dietary adaptation. Through in vitro fermentation assay, we demonstrate that microbial fermentation is context‐dependent, maintaining stability in extracting energy from leaf foods while exhibiting plasticity for non‐leaf foods. However, our findings also suggest that the gut microbiome's buffering capacity is constrained by environmental conditions. While microbial fermentation enhances the host's energy revenue, its compensatory role is inherently limited by the fermentability of available foods. By linking microbial dynamics with dietary shifts, this study contributes to a broader understanding of primate feeding ecology and the functional role of the gut microbiome in supporting primate survival in seasonal environments. Our findings underscore the need for further comparative studies across diverse ecological settings to assess how microbial plasticity facilitates the dietary adaptation of mammals, including primates.

## Author Contributions

**Wanyi Lee:** conceptualization (equal), data curation (lead), investigation (lead), visualization (lead), writing – original draft (lead). **Tianmeng He:** data curation (lead), investigation (lead), writing – review and editing (equal). **Yosuke Kurihara:** data curation (equal), investigation (equal), writing – review and editing (equal). **Izumi Shiroishi:** investigation (supporting). **Kazunari Ushida:** methodology (equal), resources (equal). **Sayaka Tsuchida:** methodology (equal), resources (equal). **Goro Hanya:** conceptualization (equal), funding acquisition (equal), supervision (lead), writing – review and editing (equal).

## Ethics Statement

The authors have nothing to report.

## Conflicts of Interest

The authors declare no conflicts of interest.

## Supporting information


**Figure S1:** (A) Observed ASVs and (B) Shannon index of the macaque gut microbiome from May 2018 to April 2019.
**Figure S2:** Compositional bar plot at (A) phyla level and (B) family level.
**Figure S3:** Correlation plot among log‐transformed dry weight intake of protein, fiber, and lipid.

**Table S1:** Number of the focal sessions and fecal samples collected for molecular experiments.
**Table S2:** Results of dbRDA analysis in weighted and unweighted UniFrac.
**Table S3:** Fecal SCFA level across seasons.

## Data Availability

Raw sequence data for the gut microbiome will be deposited in the DDBJ database under project PRJDB20590.
