# Supplementary material for: Seasonal Adaptation of the Gut Microbiome in Japanese Macaques: Linking Gut Microbiome Shifts With Fermentative Function
Source: Ecol Evol. 2025 Sep 1;15(9):e72076. doi: 10.1002/ece3.72076 (PMC12401546; doi:10.1002/ece3.72076)
Supplement: Supplementary file 1 — Figure S1: (A) Observed ASVs and (B) Shannon index of the macaque gut microbiome from May 2018 to April 2019. Figure S2: Compositional bar plot at (A) phyla level and (B) family level. Figure S3: Correlation plot among log‐transformed dry weight intake of protein, fiber, and lipid. [file ECE3-15-e72076-s002.pdf]

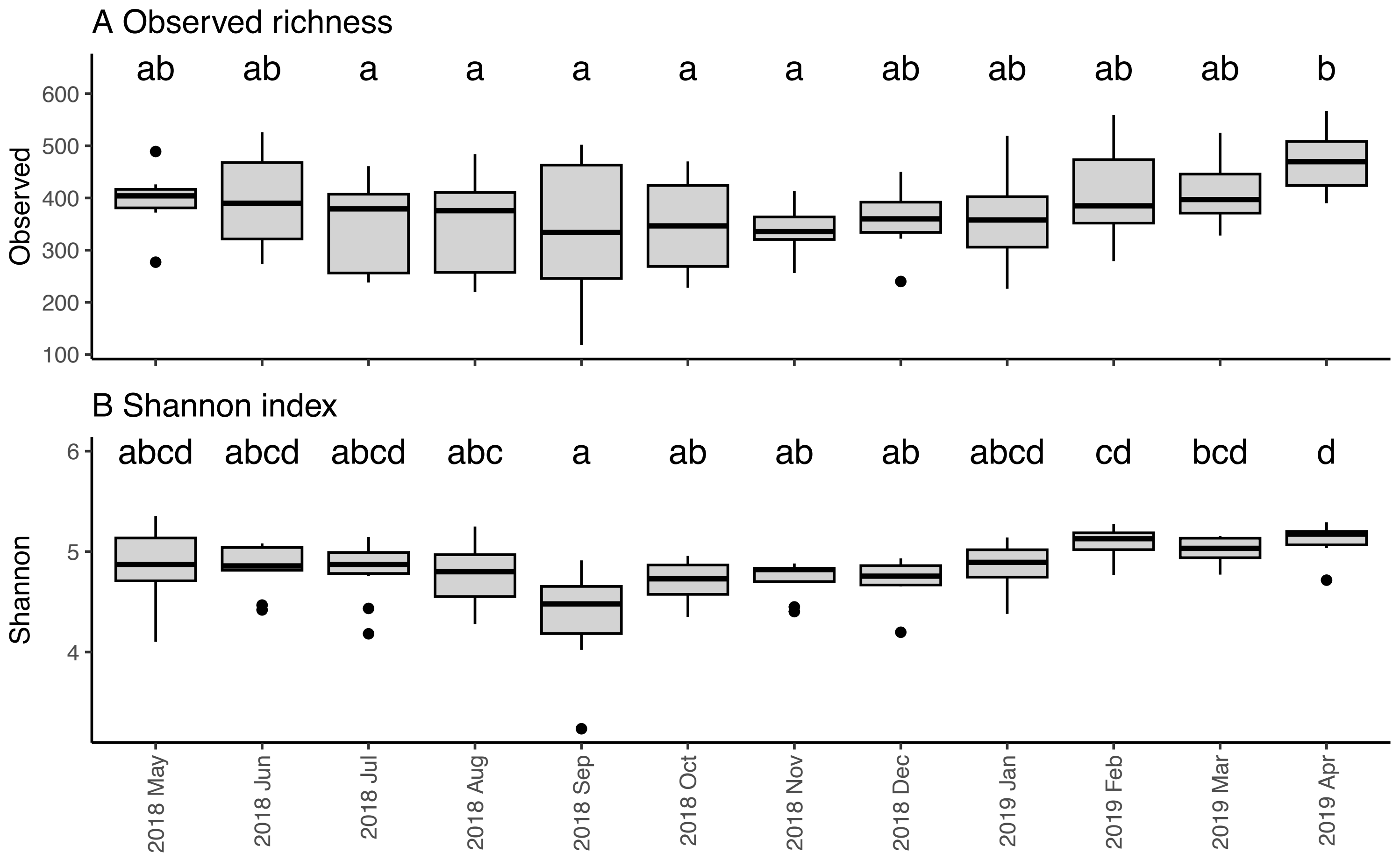

A Observed richness
ab
ab
a
a
a
a
a
ab
ab
ab
ab
b
Observed
600
500
400
300
200
100
B Shannon index
abcd
abcd
abcd
abc
a
ab
ab
ab
abcd
cd
bcd
d
Shannon
6
5
4
2018 May
2018 Jun
2018 Jul
2018 Aug
2018 Sep
2018 Oct
2018 Nov
2018 Dec
2019 Jan
2019 Feb
2019 Mar
2019 Apr

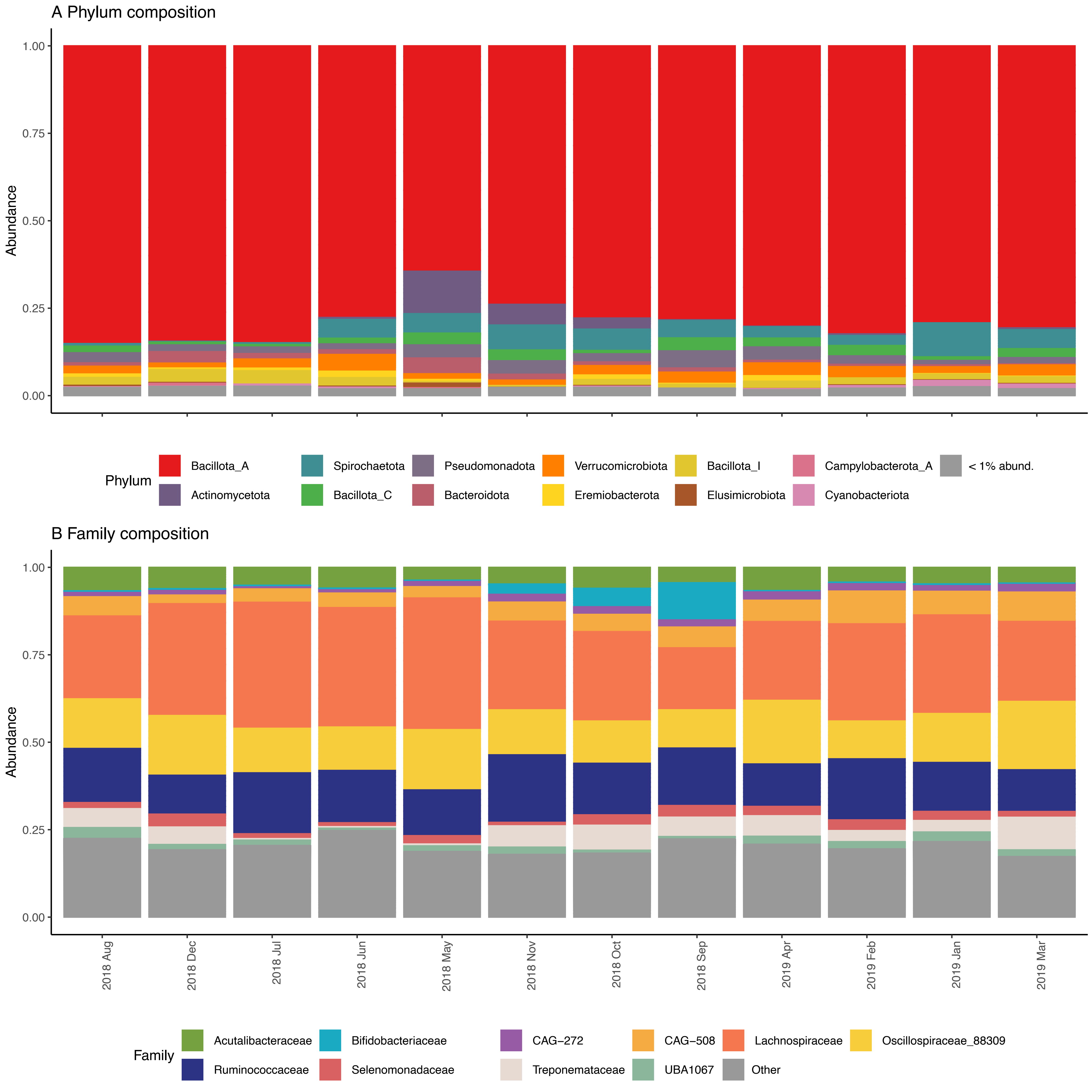

A Phylum composition
Abundance
1.00
0.75
0.50
0.25
0.00
Phylum
Bacillota_A
Actinomycetota
Spirochaetota
Bacillota_C
Pseudomonadota
Bacteroidota
Verrucomicrobiota
Eremiobacterota
Bacillota_I
Elusimicrobiota
Campylobacterota_A
Cyanobacteriota
< 1% abund.
B Family composition
Abundance
1.00
0.75
0.50
0.25
0.00
2018 Aug
2018 Dec
2018 Jul
2018 Jun
2018 May
2018 Nov
2018 Oct
2018 Sep
2019 Apr
2019 Feb
2019 Jan
2019 Mar
Family
Acutalibacteraceae
Ruminococcaceae
Bifidobacteriaceae
Selenomonadaceae
CAG−272
Treponemataceae
CAG−508
UBA1067
Lachnospiraceae
Other
Oscillospiraceae_88309

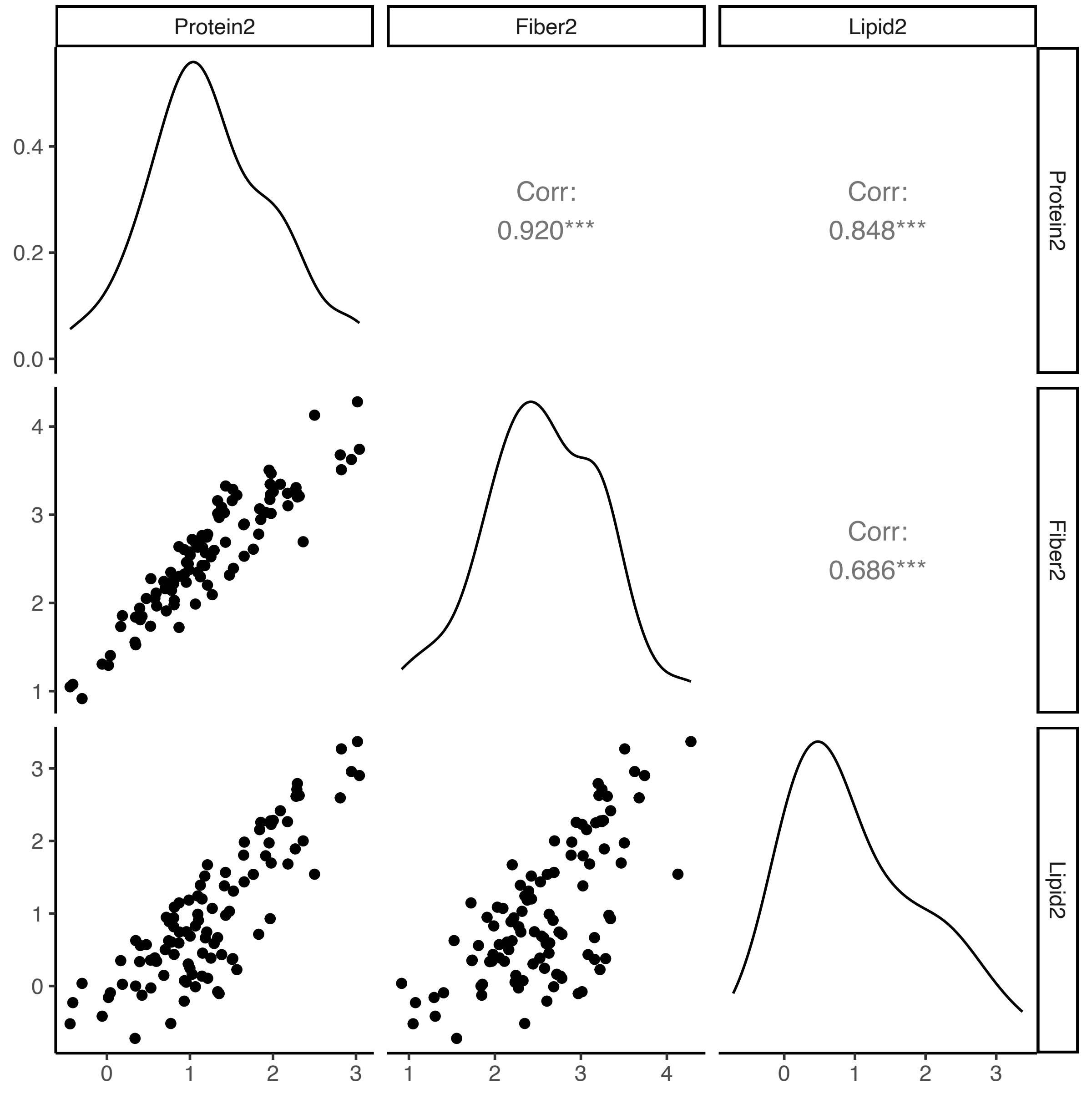

Protein2
Fiber2
Lipid2
Corr:
0.920***
Corr:
0.848***
Corr:
0.686***
0.0
0.2
0.4
1
2
3
4
0
1
2
3
0
1
2
3
1
2
3
4
0
1
2
3

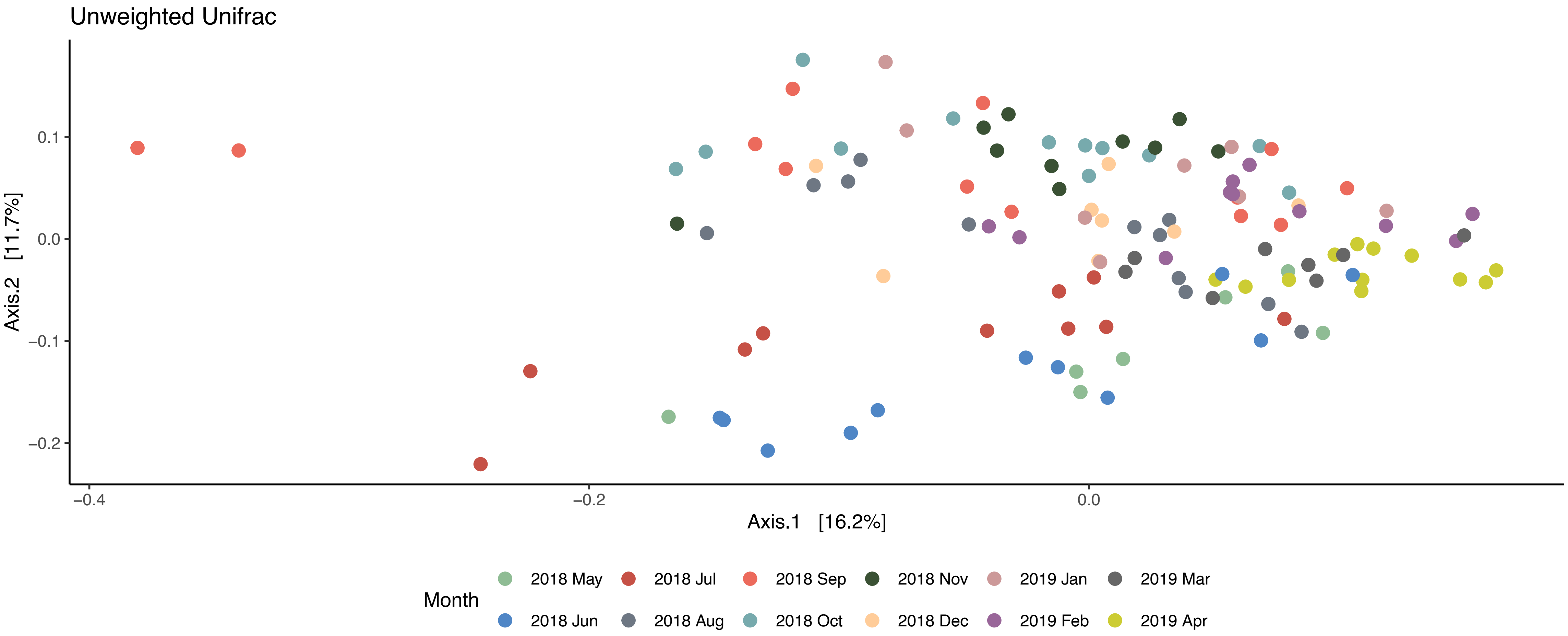
Unweighted Unifrac
0.1
0.0
−0.1
−0.2
Axis.2 [11.7%]
−0.4
−0.2
0.0
Axis.1 [16.2%]
Month
2018 May
2018 Jun
2018 Jul
2018 Aug
2018 Sep
2018 Oct
2018 Nov
2018 Dec
2019 Jan
2019 Feb
2019 Mar
2019 Apr

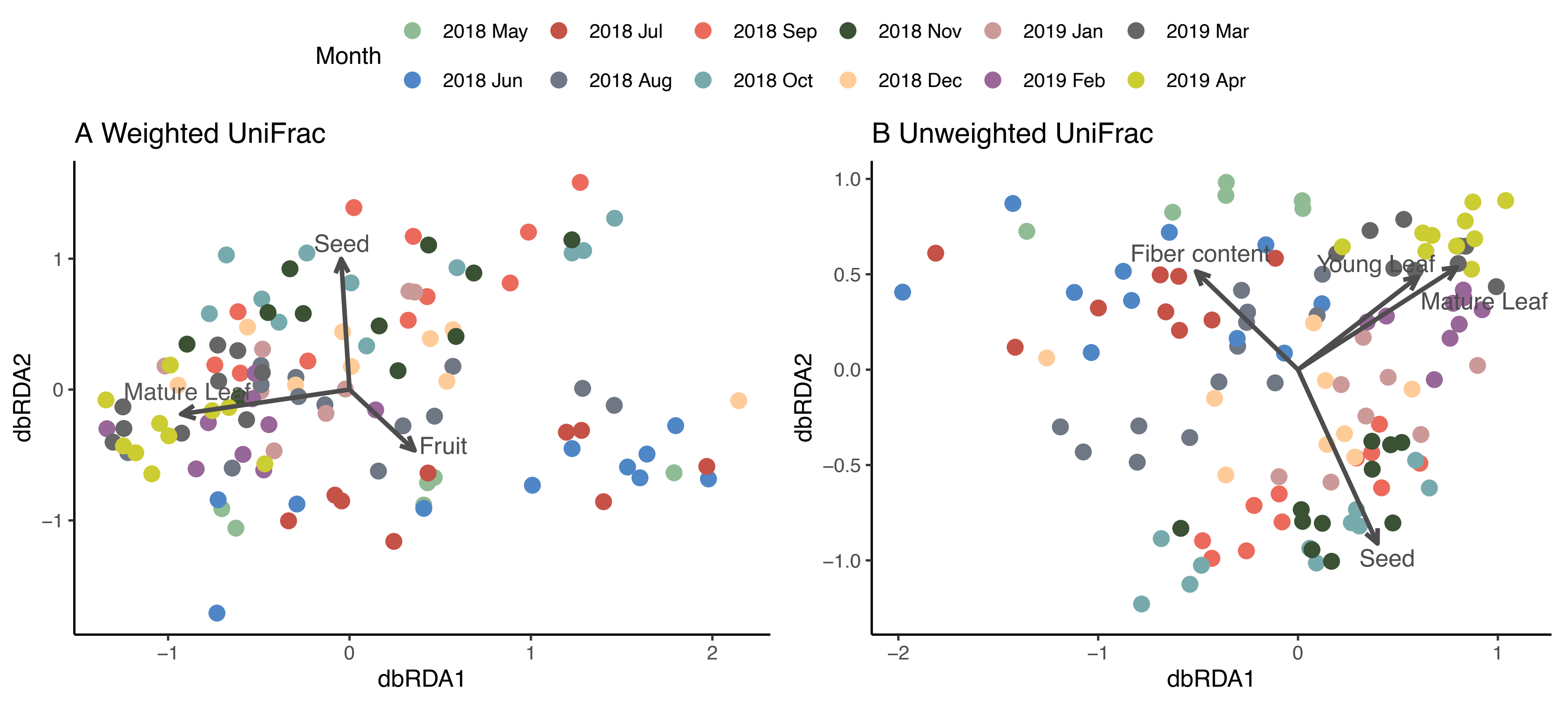

Month
2018 May
2018 Jul
2018 Sep
2018 Nov
2019 Jan
2019 Mar
2018 Jun
2018 Aug
2018 Oct
2018 Dec
2019 Feb
2019 Apr
A Weighted UniFrac
Seed
Mature Leaf
Fruit
dbRDA1
dbRDA2
B Unweighted UniFrac
Fiber content
Young Leaf
Mature Leaf
Seed
dbRDA1
dbRDA2
